# Improved Sheet Resistance of Nanofiber-Based Transparent Conducting Electrodes Using Silver Nanowires

**DOI:** 10.3390/polym13213856

**Published:** 2021-11-08

**Authors:** Sujin Cha, Byeolyi Choi, Eugene Lee, Gilsoo Cho

**Affiliations:** Department of Clothing & Textiles, Yonsei University, Seoul 33600, Korea; sj765000@yonsei.ac.kr (S.C.); byul1230@yonsei.ac.kr (B.C.); gscho@yonsei.ac.kr (G.C.)

**Keywords:** transparent conducting electrodes, polyvinylidene fluoride nanofiber web, silver nanowires, poly(3,4-ethylenedioxythiophene):poly(styrene sulfonate), smart textiles

## Abstract

There is an increased need for research on flexible transparent electrodes (FTEs) because they are critical to next-generation electronic devices, such as wearable computers. In this study, highly conductive transparent conducting electrodes, based on polyvinylidene fluoride (PVDF) nanofiber webs treated with poly(3,4-ethylenedioxythiophene):poly(styrene sulfonate) (PEDOT:PSS) and silver nanowires (AgNWs), were successfully fabricated. Transparent conducting electrodes (TCEs) were obtained by a brush-painting process using different weight ratios of a AgNWs to PEDOT:PSS solution, and the surface, electrical, optical, and chemical properties, as well as the tensile strength of the samples, were determined. It was found that the electrical conductivity of the samples improved as the AgNW content increased, but the light transmittance decreased. In this work, there was a slight decrease in the optical properties and a considerable increase in the electrical properties due to the hybridization of AgNWs and PEDOT:PSS, compared to using only PEDOT:PSS. When considering both transparency and electrical conductivity, which are essential parameters of TCEs, sample PA2, which was treated by mixing AgNWs and PEDOT:PSS/dimethyl sulfoxide (DMSO) in a ratio of 1:5 (16.67 wt% of AgNWs), was found to be the best sample, with a sheet resistance of 905 Ω/cm^2^ and light transmittance of 79%.

## 1. Introduction

Smart clothing is capable of sensing and responding to stimuli from the environment and the wearer via the inclusion of smart textiles. The clothing provides electrical, thermal, mechanical, chemical, or magnetic stimuli in response to external stimuli. Smart textiles incorporate electronic devices while maintaining fiber-specific properties [1]. They collect information about external stimuli and adapt to the environment and environmental conditions [2].

As the interest in wearable electronic devices grows, electronic devices have acquired new characteristics, such as flexibility and transparency. Therefore, the need for research on FTEs has increased, as they play an important role in next-generation electronic devices, e.g., wearable computers, organic photovoltaics (OPVs), organic light-emitting diodes (OLEDs), and displays [3]. Varying levels of the key parameters of transparent conducting electrodes (TCEs) (including sheet resistance and optical transmittance) are required. These depend on the type of device to which they are applied. Generally, more than 80% of light should be transmitted in the visible light area (visible ray area), and the sheet resistance should be less than 100 Ω/cm^2^ [4,5].

Indium tin oxide (ITO) is the most commonly used material for manufacturing TCEs because of its high optoelectronic performance [6]. However, ITO TCEs are unsuitable for smart-textile or smart-clothing applications [7]. Research on alternative materials with mechanical flexibility and optical transparency is actively underway; silver nanowires (AgNWs), [8] carbon nanotubes (CNTs), [9] graphene, [10] and conductive polymers are used as materials for TCEs [11].

AgNWs have a high aspect ratio and surface area in the form of randomly distributed thin wires with a nanometer-scale diameter in cross-section that exhibit optical and electrical properties similar to those of ITO; they require a simple treatment process and have superior flexibility [12,13]. However, AgNWs have inherent problems, e.g., a random network structure that is easily oxidized in air, low chemical stability, and decreased light transmittance as the density increases, resulting in haziness [3,14]. Carbon allotropes, e.g., CNTs and graphene, can enhance the flexibility of TCEs but are limited by low conductivity and difficulties with forming large-scale uniform films [15].

Intrinsically conducting polymers (ICPs) are inherently conductive polymers with a covalent structure, in which single and double carbon bonds repeat alternately in the backbone making them conductive by π-electron density delocalization, that can increase electrical conductivity by secondary doping with solvents [16]. Representative ICPs include polyaniline (PANI) [17,18,19,20,21,22], polypyrrole (PPy) [23], and polythiophene (PTh) [24]. PEDOT is highly conductive and stable; therefore, it is utilized in various fields [13]. PEDOT:PSS is a synthetic poly(3,4-ethylenedioxythiophene) (PEDOT) and poly(styrenesulfonic acid) (PSS) polymer that can be dispersed in water. It is flexible, has high light transmittance, solution processing, and thermal stability, but has a lower conductivity than ITO. However, adding solvents such as ethylene glycol, glycerol, dimethyl sulfoxide (DMSO), and sorbitol improves its morphology and conductivity [25]. When adding DMSO to PEDOT:PSS, some parts of the insulator PSS chains found around the conductive polymer PEDOT chains were removed, and the electrical properties improved because of the increased contact [26]. Thus, PEDOT:PSS has received considerable attention as a substitute material for ITO [27].

Studies have been conducted on hybrid composites of different materials to compensate for the limitations of the individual materials by enhancing their physical properties while maintaining their respective advantages. There has been research on hybrid TCEs. Wei et al. manufactured TCEs with a low sheet resistance of 12 Ω/cm^2^ and 83% light transmittance by coating AgNWs and PEDOT:PSS using a hot-pressing method [28]. Li et al. produced a flexible composite TCE with a low sheet resistance of 9.4 Ω/cm^2^ and a light transmittance of 89.2% by spin-coating PEDOT:PSS and AgNWs on polyethylene terephthalate [29]. However, research on the production of nanofiber-web-based TCEs is still lacking. To fill this gap, this study aims to manufacture TCEs with excellent electrical and optical properties by mixing PEDOT: PSS and AgNWs, then coating the nanofiber webs with the mixture.

The nanofiber web, a non-woven textile made of nanofibers with a thickness of tens to hundreds of nanofibers, is a high-tech material with a large surface area to volume ratio and excellent lightness and flexibility [30]. Therefore, nanofiber webs, particularly nanofiber-web-based textile sensors that can measure biological signals, e.g., electrocardiogram (ECG) and strain sensors, are actively used in smart textile research [12,31,32]. Nanofiber webs are made from various fiber polymer materials, including polyurethane (PU), polyamide (PA6, PA66), polyacrylonitrile (PAN), and polyvinylidene fluoride (PVDF). PVDF exhibits favorable mechanical, pyroelectric, and piezoelectric properties; hence, it has attracted attention in a myriad of research fields [33]. PVDF is a representative semi-crystalline polymer that has three possible polymorphs (alpha, beta, and gamma phases), in accordance with the chain conformation [34]. Its polymer structure repeats the -CH_2_-CF_2_- group, [35] and has excellent heat resistance, durability, and weather resistance. It has chemical and optical stability, owing to its regular binding and high fluoride content. Typically, PVDF appears opaque due to the occurrence of diffuse reflection at the boundary between its crystalline and amorphous regions, but it can be made transparent by controlling its crystallinity; this involves distorting the structure of the C-F dipole using DMSO solvent and heat treatment [36]. This study mixed PEDOT:PSS with AgNWs using a PVDF nanofiber web as a substrate for TCEs, providing the advantages of sunlight resistance, flexibility, and a large surface area.

We previously [37] fabricated PVDF nanofiber-web-based TCEs for photovoltaic textiles with 83% light transmittance and 1496 Ω/cm^2^ sheet resistance by mixing PEDOT:PSS and DMSO. In this study, we aim to improve the conductivity of PVDF nanofiber-web-based TCEs for a photovoltaic textile using a composite of AgNWs and PEDOT:PSS/DMSO. We report an approach to improve the electrical conductivity of TCEs by using AgNWs and PEDOT:PSS/DMSO solutions with different weight ratios than those used previously [37] and by treating them on the PVDF nanofiber web using a brush-painting technique, which has advantages such as simplicity, cost efficiency, and processability. Consequently, this study shows flexible TCEs based on AgNW/PEDOT:PSS/PVDF nanofiber webs, and analyzes the surface, electrical, optical, and chemical properties, as well as the tensile strength of the AgNW/PEDOT:PSS/PVDF nanofiber web, according to the solution ratio of AgNWs to PEDOT:PSS.

## 2. Materials and Methods 

### 2.1. Materials

The textile substrate was a PVDF nanofiber web (Pardam, s.r.o. Ltd., Pardubice, The Czech Republic) manufactured by centrifugal spinning. The nanofiber diameters ranged from 200 to 500 nm, and the weight was 4.3 g/m^2^. To impart electrical conductivity to the PVDF textile substrate, 1 wt% AgNW dispersed in ethanol (which was fabricated by using a polyol synthesis process (KLK Co., Seoul, Korea) and with an average length and diameter of, respectively, 22.5 (±2.5) μm and 32.5 (±2.5) nm), 1.3 wt% poly(3,4-ethylenedioxythiophene):poly(styrenesulfonate) (PEDOT:PSS) dispersed in an aqueous solution (Sigma-Aldrich, St. Louis, MO, USA) which contented 0.5 wt% of PEDOT, 0.8 wt% of PSS, and 99.9% DMSO solution (Duksan Pure Chemicals, Ansan, Korea), were used.

### 2.2. Fabrication of Transparent Conducting Electrodes

We conducted a pretest with 1.0, 1.5, and 2.0 wt% AgNW, and these samples exhibited a low light transmittance of less than 65%. Therefore, we used 0.5 wt% AgNW in this study. To obtain a 0.5 wt% AgNW solution, 1 wt% AgNW solution from KLK Co. was diluted in ethyl alcohol.

To prepare the AgNW/PEDOT:PSS/DMSO mixed solutions, 0.5 wt% AgNW solution and PEDOT:PSS/DMSO (mixed in a ratio of 3:7) solutions were mixed by vortexing in varying ratios. Six solutions were prepared (Table 1).

**Figure 1:**
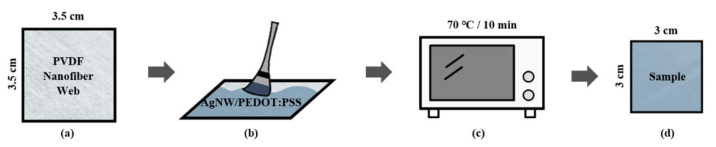
Schematic of the sample-fabrication process: (**a**) sample preparation; (**b**) brush-painting; (**c**) heat treatment; (**d**) final sample.

Figure 1 shows the electrode fabrication process. A PVDF nanofiber web (3.5 × 3.5 cm^2^) was prepared (Figure 1a) and treated with the AgNW/PEDOT:PSS/DMSO solution (100 μL) using the brush-painting method (Figure 1b). The sample was then dried for 10 min in a vacuum oven (OV-11, JEIO Tech. co., LTD, Daejeon, Korea) at 70 °C (Figure 1c). Finally, the treated sample was cut to a size of 3 × 3 cm^2^ to obtain a subsample that was uniformly treated with the solution (Figure 1d).

### 2.3. Measurements

The sheet resistances of the samples were measured five times using a four-point probe (CMT-SR 1000N, AIT, Guro-gu, Korea). The transmittance of the samples in the visible light region (400–700 nm) was measured thrice using a UV-vis spectrophotometer (V-650, JASCO Corporation, Tokyo, Japan). The surface morphology and microstructure of the samples were characterized using field-emission scanning electron microscopy (FE-SEM, JEOL-7800F, JEOL Ltd., Akishima-shi, Japan) at 2000× magnification. The thickness of the samples was measured using a surface profiler (DektakXT Stylus Profiler, Bruker Corporation, Billerica, MA, USA). X-ray photoelectron spectroscopy (XPS, K-alpha, Thermo Fisher Scientific, Waltham, MA, USA) was used to characterize the chemical properties of the samples. The tensile properties of the samples were measured using a tensile testing machine (Instron Model 34SC-1, Illinois Tool Works Inc., Glenview, IL, USA). The Kruskal–Wallis test, which is a non-parametric statistical method, was performed using IBM SPSS Statistics 25 (IBM Corporation, Armonk, NY, USA,) to investigate the effect of the AgNW treatment ratio on the sheet resistance of the samples. 

## 3. Results

### 3.1. Surface Properties of the Samples

**Figure 2:**
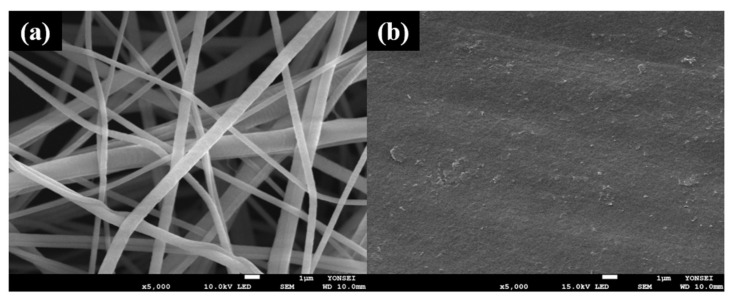

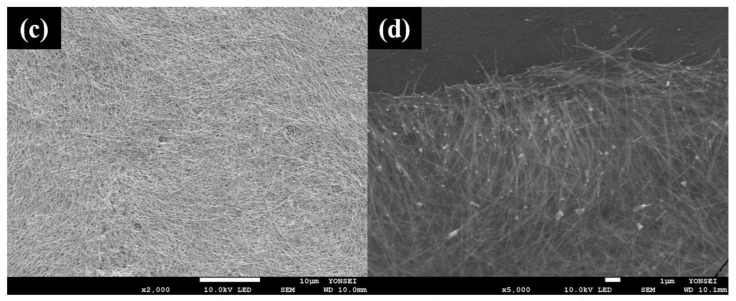
FE-SEM images of: (**a**) untreated PVDF nanofiber web (×5000); (**b**) reference sample (×5000); (**c**) AgNWs (×2000); (**d**) AgNWs (×5000).

FE-SEM was conducted to observe the changes in the surface microstructure of the samples. The surface morphologies of the untreated PVDF nanofiber web, reference sample [37] of the previous study, the AgNWs solution, and six samples (PA1–PA6) were studied. Numerous strands of nanofibers were confirmed to intertwine irregularly on the untreated PVDF nanofiber web (Figure 2a). In the reference sample (Figure 2b), the PEDOT:PSS/DMSO solution completely covered the surface of the textile with a smooth and flat coating, without visible fiber strands. In the AgNWs solution images at different magnifications, it was observed that innumerable rod-shaped silver nanowires were entangled (Figure 2c), and silver particles were also observed together with the rods (Figure 2d).

**Figure 3:**
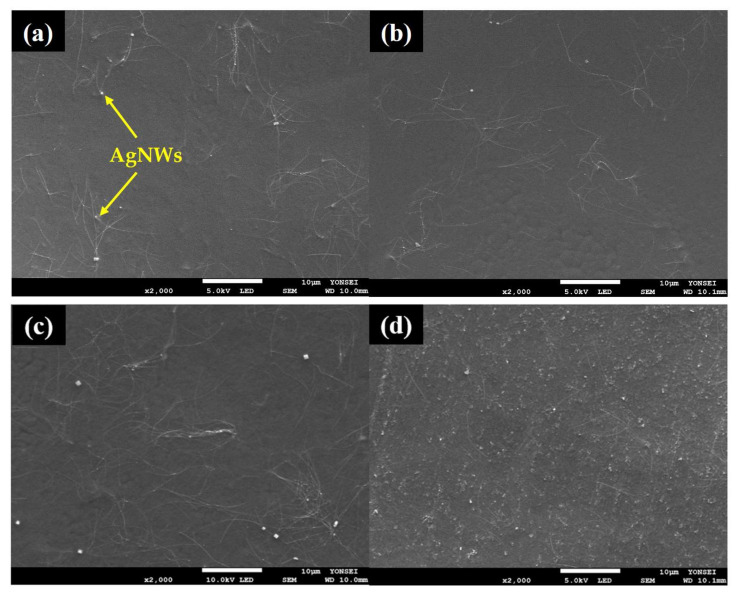

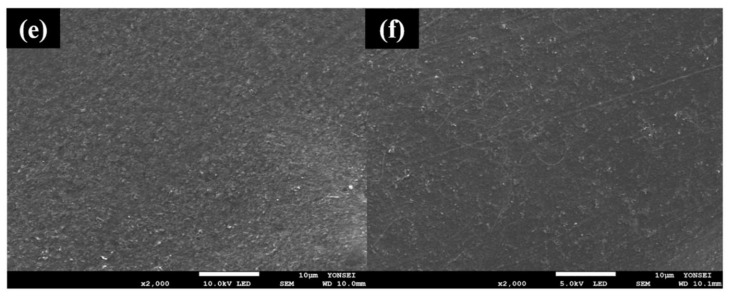
FE-SEM images of the samples (×2000): (**a**) PA1; (**b**) PA2; (**c**) PA3; (**d**) PA4; (**e**) PA5; (**f**) PA6.

The surface morphologies of samples PA1–PA6 (Figure 3a–f) showed that the conductive solution completely covered the PVDF nanofiber web. The surfaces of PA1, PA2, and PA3 looked smooth and evenly treated. Additionally, thin and long rod-shaped AgNWs could be well observed in these images. In contrast, as the treatment ratio of the AgNWs increased, the surfaces of PA4, PA5, and PA6 appeared rough and bumpy, and the fine AgNWs rods were relatively less observed. The roughness of the surface increased compared to that of the sample treated with only PEDOT:PSS, and the electrical properties of the samples improved significantly. Therefore, the surface roughness of the samples might affect the light transmittance of the samples by causing a diffuse reflection, which is consistent with the optical properties determined in this study.

The surface profiler analysis was conducted to measure and compare the thickness of the samples. The average thicknesses of the untreated PVDF nanofiber web and the reference sample were determined to be 31.37 (±2.00) and 2.93 (±0.27) μm. The average thickness of the manufactured samples (PA1–PA6) was 21.51 (±11.36), 23.61 (±4.35), 29.1 (±15.89), 31.57 (±11.55), 38.21 (±8.96), and 43.28 (±12.98) μm, respectively. The greater the quantity of silver nanowires in the sample, the greater the sample thickness; samples treated with less silver nanowires (PA1, PA2, and PA3) exhibited a decreased thickness compared to that of the PVDF nanofiber web. Samples PA4, PA5, and PA6 exhibited increased thickness. In addition, the samples from PA1 to PA6 exhibited light transmittances of 79%, 79%, 75%, 75%, 69%, and 61%, respectively; that is, light transmittance decreased as the thickness increased.

The thickness of the samples PA1, PA2, and PA3 decreased because of the shear stress caused between the brush and the sample in the brush-painting process [38]. The nanofiber was pressed by a brush, and the thickness was reduced. Additionally, processing large DMSO quantities would have affected the reduction in thickness as the PEDOT:PSS polymer particles were removed. However, the thickness of the samples PA4, PA5, and PA6 increased compared to that of the PVDF nanofiber web, and this affected the density increase in the silver nanowires in the network structure [7]. Therefore, the thickness of the manufactured samples had a direct effect on light transmittance, as the light transmittance decreased when the added ratio of silver nanowires in the samples increased.

### 3.2. Electrical and Optical Properties of the Samples

**Figure 4:**
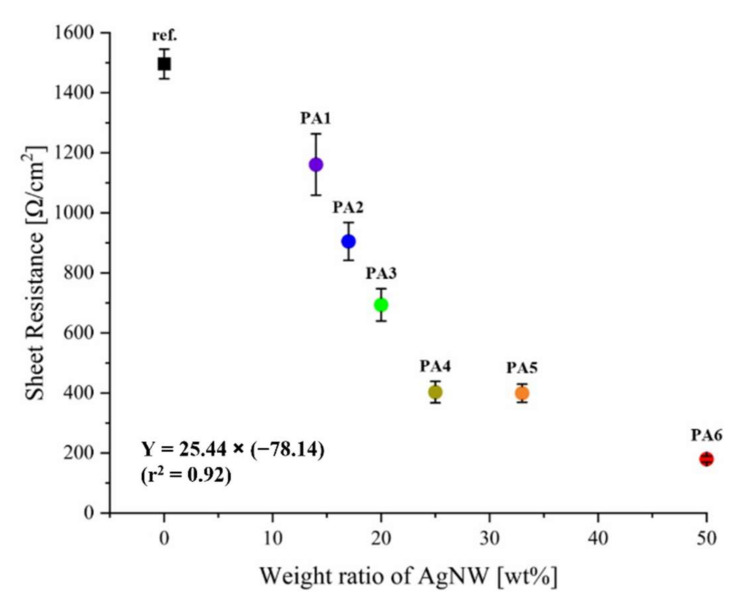
Sheet resistance of the samples as a ratio of AgNW to PEDOT:PSS.

Figure 4 and Table 2 illustrates the sheet resistance of the reference sample and the six samples. The reference sample [37] had a sheet resistance of 1496 (±129) Ω/cm^2^. The samples (PA1–PA6) exhibited sheet resistances ranging from 180 to 1161 Ω/cm^2^. Compared to that of the reference sample, the sheet resistance of the other samples decreased by a minimum of 1.3 times and a maximum of 8.3 times as the proportion of AgNWs increased. Silver is a metal with a high electrical conductivity. Therefore, when used in a mixture of AgNW and PEDOT:PSS, it is expected to have a noticeable effect on the conductivity. The structure of the AgNWs also affects their conductivity. A previous study [39] found that the silver nanowires were longer when compared with Ag particles; therefore, when nanowires were used as a composite, the conductivity improved because they formed more connection paths. Additionally, PEDOT:PSS provided an electron pathway in the non-crossed parts of AgNWs with a random network structure, forming a uniform surface in the samples. Consequently, the electrical performance of the samples improved using this method [6].

The sheet resistances of samples PA1–PA6 were compared (Figure 4). PA1, treated with PEDOT:PSS/DMSO and AgNW at a 1:1/6 weight ratio, had the highest sheet resistance at 1161 (±228) Ω/cm^2^, and sample PA6, treated with PEDOT:PSS/DMSO and AgNW at a 1:1 weight ratio, had the lowest sheet resistance at 180 (±22) Ω/cm^2^. PA2, PA3, PA4, and PA5 exhibited sheet resistances of 905 (±141), 694 (±120), 403 (±80), and 399 (±67) Ω/cm^2^, respectively. The sheet resistance decreased gradually as the weight ratio of AgNW to the PEDOT:PSS/DMSO solution increased. Thus, sample PA6, containing the highest mixing ratio of AgNW, had the lowest electrical resistance and exhibited the best electrical performance. In addition, as a result of the statistical analysis, the p-value of the sheet resistance of the samples was 0.000, indicating that there was a significant difference below the significance level *p* < 0.001, and it could be seen that the treatment ratio of AgNWs directly affects the sheet resistance of the samples.

**Figure 5:**
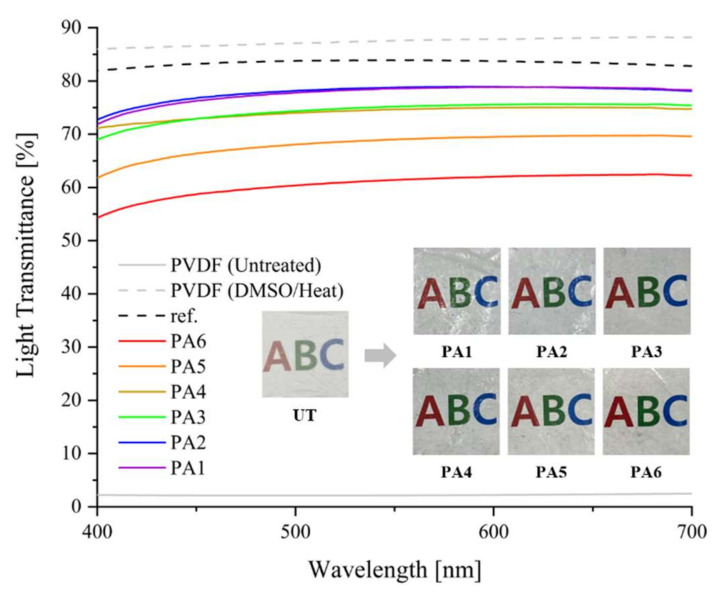
Light transmittance of UT and the samples as a ratio of AgNW to PEDOT:PSS.

Light transmittance in the visible region (400–700 nm) was measured to examine the optical properties of the samples. Figure 5 illustrates that the untreated PVDF nanofiber web had a 2% transmittance and that the PVDF nanofiber web treated with DMSO and heat treatment had an 88% transmittance at 550 nm. The samples PA1–PA6 exhibited light transmittances of 79%, 79%, 75%, 75%, 69%, and 61%, respectively.

The light transmittance gradually increased as the AgNW content decreased from 1:1 (PA6) to 1:1/6 (PA1); thus, the maximum light transmittance of the samples was 79%. A previous study [40] states that AgNW-treated samples have rough surfaces because of the unique metallic properties of AgNWs, which cause severe diffuse reflections of light. That is, the higher the AgNW content, the lower the light transmittance of the samples. However, in this study, the AgNW was mixed with the PEDOT:PSS/DMSO solution to fill in the gaps in the AgNW networks, thereby smoothening the surface of the samples and improving their light transmittance.

However, there was no noticeable difference in light transmittance between samples PA1 and PA2, which exhibited the best optical properties (79%). PA1 and PA2 samples exhibited excellent light transmittance of nearly 80% and had similar optical properties to the sample (83%) in the previous study [37].

**Figure 6:**
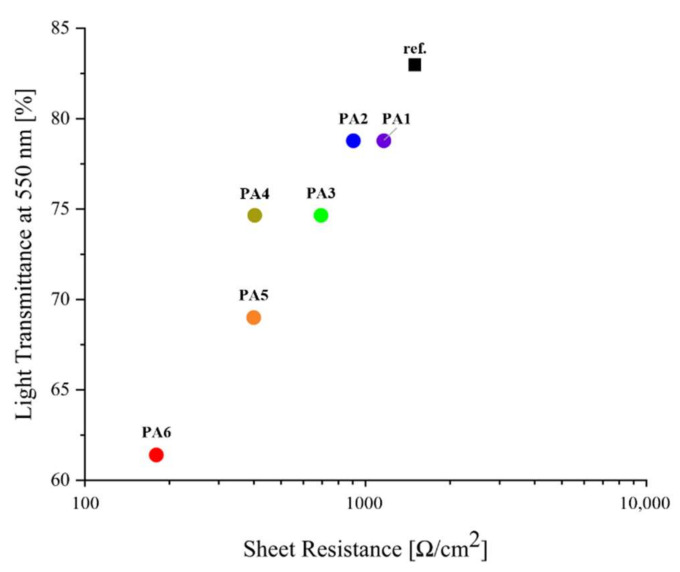
Electrical and optical characteristics of the samples as a ratio of AgNW to PEDOT:PSS.

Figure 6 shows a plot of the transmittance of the flexible TCEs at 550 nm versus their sheet resistance. Unlike the reference sample treated with only PEDOT:PSS/DMSO [37], the samples treated with solutions of PEDOT:PSS/DMSO and AgNW exhibited better electrical conductivity and light transmittance that reduced rapidly compared to the reference sample because of the addition of opaque metal. However, PA1 (14.29 wt% AgNWs) and PA2 (16.67 wt% AgNWs) exhibited better electrical conductivity than that of the reference sample [37], and a light transmittance close to 80%. Considering both the electrical and optical properties of the TCEs, sample PA2 exhibited electrical properties under 1000 Ω/cm^2^, demonstrating better electrical properties than that of the reference sample [37], and a light transmittance close to that (80%) required of TCEs. Hence, PA2 demonstrated the best performance in this study.

### 3.3. Chemical Properties of the Samples

**Figure 7:**
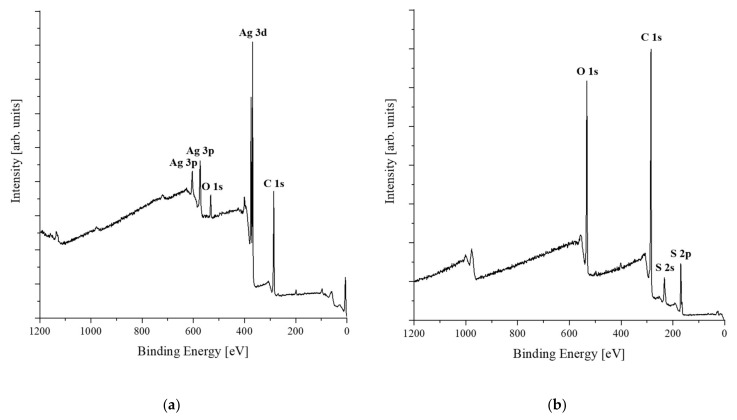

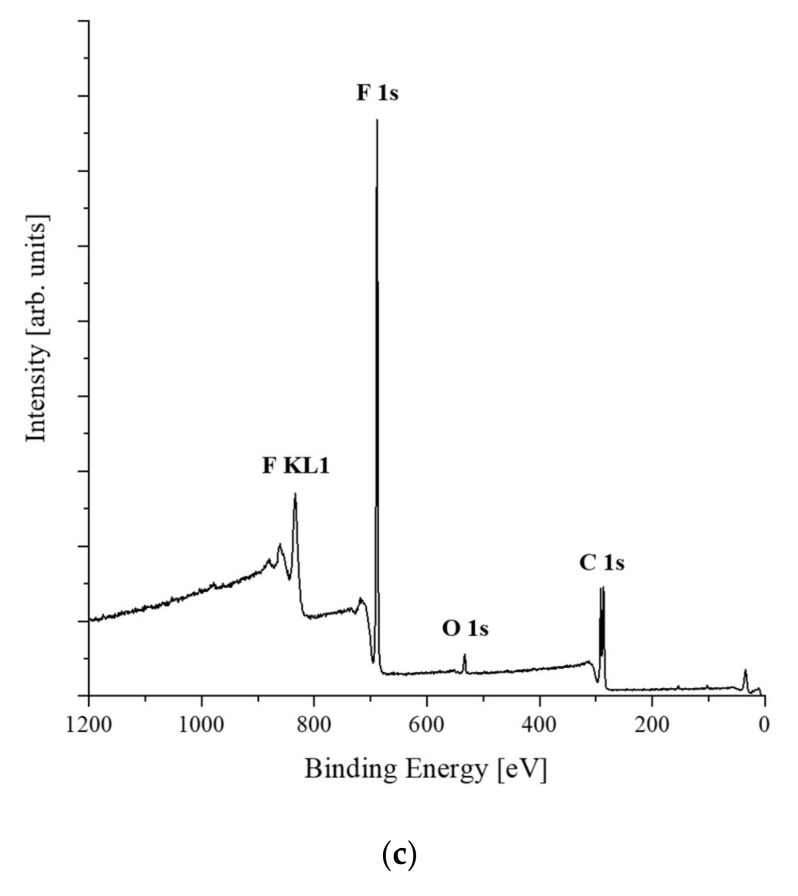
XPS profiles of: (**a**) AgNW; (**b**) PEDOT:PSS; (**c**) PVDF nanofiber web.

XPS was conducted to examine the chemical properties of the samples. Figure 7 illustrates the results of the XPS of the AgNW solution (Figure 7a), the PEDOT:PSS solution (Figure 7b), and the PVDF nanofiber web (Figure 7c). Ag 3p (603 eV, 573 eV) and Ag 3d (368 eV) peaks corresponding to AgNWs; C 1s (285 eV), S 2s (233 eV), and S 2p (169 eV) peaks corresponding to PEDOT:PSS; and F KL1 (833 eV), F 1s (688 eV), and C 1s (286 eV) peaks corresponding to the PVDF nanofiber web were observed.

**Figure 8:**
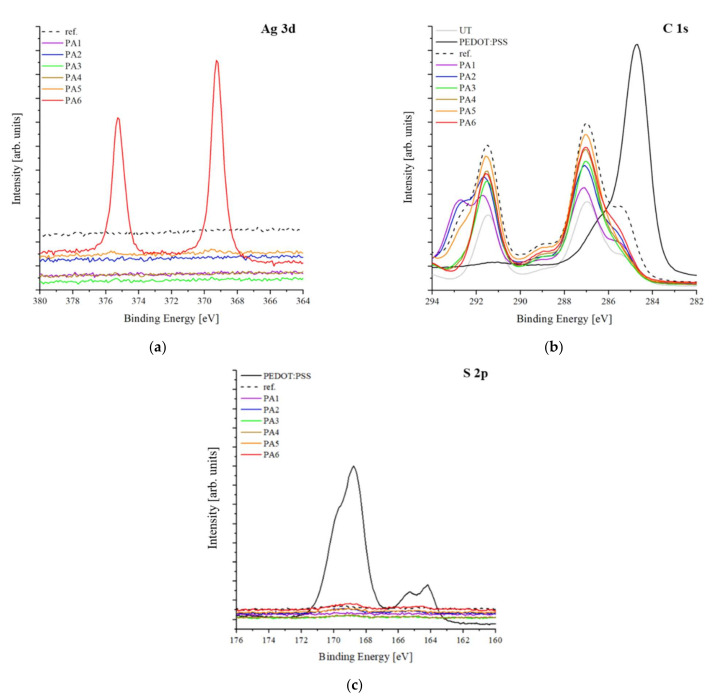
XPS profiles of samples treated with AgNW/PEDOT:PSS solution: (**a**) Ag 3d; (**b**) C 1s; (**c**) S 2p core-level spectra.

Figure 8 displays the XPS results for the samples. In samples PA1–PA5, there was no peak corresponding to Ag 3d since samples PA1-PA5 were sparsely coated with very small amounts of AgNW; only sample PA6 (AgNW:(PEDOT:PSS) = 1:1) (treated with the largest proportion of AgNWs) exhibited Ag 3d 5/2 and Ag 3d 3/2 peaks at 368.1 eV and 374.3 eV, respectively (Figure 8a). [40] All samples were similar to the reference sample in Figure 8b, and peaks corresponding to CF_2_ (290.9 eV) and CH_2_ (286.5 eV) of the PVDF nanofiber web (UT) were commonly observed. [41] However, elemental C corresponding to PEDOT:PSS was not observed. Previous studies have mentioned [37,42] that when PEDOT:PSS was doped with organic solvents, the PSS shell surrounding the PEDOT polymer was partially removed. However, when excess DMSO was used, both the PSS and PEDOT particles were removed. In this study, elemental C was not observed because many PEDOT and PSS particles were removed by the large quantity of DMSO. Elemental S corresponding to the PSS polymers of PEDOT:PSS was not found in either the reference sample or the samples used in this study (Figure 8c). This is because a large quantity of DMSO was processed and the PSS polymers were removed.

### 3.4. Tensile Properties of the Samples

Tensile strength tests were conducted to determine the changes in tensile properties when the PVDF nanofiber web was manufactured with TCE via the treatment of conductive materials mixed with AgNWs and PEDOT:PSS. The untreated PVDF nanofiber web (UT) and the PA2 sample exhibiting the highest performance were tested. The results (measured five times per sample) are presented in Table 3. The average tensile strength of the UT and PA2 was 3.12 (±0.27) and 4.88 (±0.51) MPa, respectively. Although PA2 was thinner than the UT, the tensile strength increased. The elongation of PA2 decreased as the PVDF nanofiber web changed from a tangled web structure on the surface to the film form as it was coated with the conductive solution (FE-SEM results), but the initial modulus of elasticity was high, and it could be inferred that the tensile strength of PA2 increased accordingly. The strength of the UT was relatively low, because when the yield point, which was the point at which the cracks occurred, the UT was generated with a more random structure of fibers. This is thought to be because the cohesion between the fibers is lowered in a thin film such as TCE, and cracks can occur between the nanofiber structures more readily than when the surface is flat and dense and consists of a single sheet. However, the displacement length of the UT was 11.92 (±0.67) nm, which was higher than that of PA2 (5.54 (±1.43)) nm, indicating that the elongation and flexibility of the UT was better than PA2. Additionally, the tensile strain, according to the displacement length of UT, was 30.53 (±4.08%), higher than that of PA2 (15.53 ± 1.69%).

As a result, when the nanofiber web was processed with a transparent electrode such as PA2, the web structure changed into a uniform sheet structure. The tensile force was applied evenly throughout the film, increasing the ability to withstand it instantaneously, but the displacement length was shorter than that of the UT; therefore, the elongation was lower.

## 4. Conclusions

We used AgNWs to improve the electrical conductivity of fiber-based TCEs. The effects of different weight ratios of PEDOT:PSS/DMSO and AgNWs were confirmed by investigating the surface, electrical, optical, chemical, and tensile properties of the samples. Consequently, the electrical conductivity of the specimens increased as the AgNW content increased, but the light transmittance decreased. 

We manufactured a textile-based TCE with a composite by mixing AgNW and PEDOT:PSS. Sample PA2 (sheet resistance 905 Ω/cm^2^ and 79% light transmittance), fabricated with a solution mixed with AgNWs and PEDOT:PSS/DMSO (1:5, 16.67 wt% AgNWs), exhibited the best performance and, compared to the previous study, the electrical conductivity was significantly improved and the light transmittance was also close to 80%, proving its availability as a TCE. However, the electrical characteristics of the TCEs varied depending on the final application. Therefore, further studies should be conducted to develop fiber-based TCEs that can replace glass-based TCEs. Furthermore, studies are needed to confirm whether TCEs perform adequately by applying fabricated fiber-based TCEs to OPVs, OLEDs, and displays.

## Figures and Tables

**Table 1:** Characteristics of the untreated PVDF nanofiber web (UT) and samples.

Sample Name	Ratio of PEDOT:PSS/DMSO to AgNW		Weight Ratio of AgNW (wt%)
	PEDOT:PSS/DMSO	AgNW	
UT	-	-	-
Reference	1	-	00.00
PA1	1	1/6	14.29
PA2	1	1/5	16.67
PA3	1	1/4	20.00
PA4	1	1/3	25.00
PA5	1	1/2	33.33
PA6	1	1	50.00

**Table 2:** Sheet resistance, light transmittance and thickness of samples.

Sample Name	Sheet Resistance (Ω/cm^2^)	Light Transmittance (%)	Thickness (μm)
PA6	180 (±22)	61	43.28 (±12.98)
PA5	399 (±67)	69	38.21 (±8.96)
PA4	403 (±80)	75	31.57 (±11.55)
PA3	694 (±120)	75	29.1 (±15.89)
PA2	905 (±141)	79	23.61 (±4.35)
PA1	1161 (±228)	79	21.51 (±11.36)
UT	-	2	31.37 (±2.00)

**Table 3:** Tensile properties of the samples (UT, PA2).

Sample	UT	PA2
Tensile stress (MPa)	3.12 (±0.27)	4.88 (±0.51)
Tensile displacement at break (nm)	11.92 (±0.67)	5.54 (±1.43)
Tensile strain (%)	30.53 (±4.08)	15.53 (±1.69)

## Data Availability

Not applicable.
